# Newly diagnosed HIV patients in Lima, Peru: a comparison of individuals diagnosed through an intervention program versus self-referred individuals

**DOI:** 10.1186/1758-2652-13-S4-P171

**Published:** 2010-11-08

**Authors:** JA Hidalgo, N Castillo, C Agurto

**Affiliations:** 1 Via Libre, Lima, Peru

## Background

An extensive intervention program to promote awareness, HIV testing, diagnosis and access to medical care in at-risk populations was developed by Via Libre between Nov 2007 and Oct 2009 in Lima, Peru (Proyecto Somos).

## Purpose of the study

We evaluate the impact of this type of intervention on the presenting clinical conditions of newly diagnosed persons.

## Methods

Review of medical records of self-referred individuals (Group 1) and patients diagnosed through the intervention program (Group 2), and presenting for the first time to Via Libre HIV Clinic. We compared the initial condition of both groups. Cases included in the analysis completed a baseline clinical and laboratory evaluation, and had a decision made by the treating physician regarding whether HAART was required or not.

## Results

During the study period, 523 HIV-positive persons presented for initial care at our clinic. We reviewed 303 records, out of which 216 were eligible for analysis. One hundred and thirty-two corresponded to the self-referred group (Group 1) and 84 to the intervention group (Group 2). Both groups had similar mean age (35.5 yrs., Group 1; 28.8 yrs., Group 2; p:0.44), and time between diagnosis and presentation to clinical evaluation (1.8 mos., Group 1, 1.0 mos., Group 2). Mean baseline CD4+ cell count was significantly lower in Group 1 (287.6 +/- 217.6 vs. 392.56 +/- 249.8, p< 0.5). A higher number of patients in Group 1 presented with AIDS-related clinical symptoms (Group 1: 47.7%, n=63; Group 2: 28.6%, n=24), and had indication of immediate initiation of antiretroviral therapy (Group 1: 62.8%, n=83, Group 2: 48.8%, n=41).

## Conclusions

Patients diagnosed through the intervention program presented with less advanced HIV infection and had chance to earlier initiation of HAART. Intervention programs to actively diagnose HIV give a significant opportunity to provide timely medical care to HIV-infected individuals in resource-limited settings. These initiatives should be integrated to national HIV care programs.

